# Validation of the adapted Leeds sleep evaluation questionnaire in Ethiopian university students

**DOI:** 10.1186/s12955-018-0876-0

**Published:** 2018-03-13

**Authors:** Md. Dilshad Manzar, Mohammed Salahuddin, Tarekegn Tesfaye Maru, Ahmad Alghadir, Shahnawaz Anwer, Ahmed S. Bahammam, Seithikurippu R. Pandi-Perumal

**Affiliations:** 1 Department of Nursing, College of Applied Medical Sciences, Majmaah University, Al Majmaah, Kingdom of Saudi Arabia; 2 Department of Biomedical Sciences, College of Health Sciences, Mizan-Tepi University (Mizan Campus), Mizan-Aman, Ethiopia; 3 Department of Pharmacy, College of Health Sciences, Mizan-Tepi University (Mizan Campus), Mizan-Aman, Ethiopia; 4 Rehabilitation Research Chair, College of Applied Medical Sciences, King Saud University, Riyadh, Saudi Arabia; 5 The University Sleep Disorders Center, College of Medicine, King Saud University, Box 225503, Riyadh, 11324 Saudi Arabia; 6 National Plan for Science and Technology, College of Medicine, King Saud University, Riyadh, Saudi Arabia; 7 Somnogen Canada Inc, College Street, Toronto, ON Canada

**Keywords:** LSEQ, Ethiopia, Insomnia, University students, Sleep

## Abstract

**Background:**

Current evidence supports the applicability of the Leeds Sleep Evaluation Questionnaire (LSEQ) in screening for insomnia. The psychometric properties of the LSEQ have never been investigated in an African population. Therefore, this study aimed to validate the adapted version of the LSEQ-Mizan (LSEQ-M) in Ethiopian university students.

**Methods:**

Of a preliminary sample of 750 (random sampling), 424 students (age = 21.87 ± 4.13 years and body mass index = 20.84 ± 3.18 kg/m^2^) from Mizan-Tepi University, Mizan-Aman, South-west Ethiopia completed the LSEQ-M, the General Anxiety Disorder Scale-7 and a semi-structured questionnaire for socio-demographics. Insomnia was screened in accordance with the International Classification of Sleep Disorders as a measure of concurrent validity.

**Results:**

Although, individual items showed ceiling and floor effect, the LSEQ-M as a scale did not have these effects. Good internal consistency (Cronbach’s alpha of 0.84) and strong internal homogeneity as measured by the correlation coefficient between items scores and the LSEQ-M global score was found. The LSEQ-M showed excellent screening applicability for insomnia with optimal cut-off scores of 52.6 (sensitivity 94%, specificity 80%), and the area under the curve, 0.95 (*p* < 0.0001). The original 4-Factor model was valid in Ethiopian university students for screening for insomnia.

**Conclusion:**

The LSEQ-M has excellent psychometric validity in screening for insomnia among Ethiopian university students.

**Electronic supplementary material:**

The online version of this article (10.1186/s12955-018-0876-0) contains supplementary material, which is available to authorized users.

## Background

The growing endemicity of sleep disorders is becoming a health concern around the globe [1–4]. The scarcity of sleep health infrastructure along with lack of awareness about sleep health issues in developing societies are obstructing the provision of patient care [5]. Young adults in general and university students in particular are at increased risk of sleep disorders [4, 6]. Sleep problems are highly prevalent in university students in Afro-Asian countries [1–3, 7]. Most university students in Ethiopia have sleep problems associated with poor psychological health [2, 3]. Sleep problems prevail in poor psycho-physiological health conditions such as stress, anxiety, fatigue, depression, attention deficit, reduced cognitive performance, and impaired social relationships. Sleep disturbances are associated with risk-taking behavior, drowsy driving, poor academic performance, and overall poor health among young adults including university students [2, 3, 8, 9].

Sleep disturbances in Ethiopian university students are related to insomnia and its associated conditions i.e. problems in sleep onset, short sleep duration and poor sleep quality [2, 3]. Ethiopia also has high prevalence of predisposing factors for sleep disorders such as use of alcohol, Khat, and excessive use of caffeinated beverages [2, 3, 5, 7]. The situation becomes grimmer because of the limited sleep health professionals in the country [7]. Few sleep questionnaire tools have been validated in Ethiopians. More so, there is no tool, which has been comprehensively validated in Ethiopian students. The Pittsburgh Sleep Quality Index (PSQI) was found to have adequate measures of psychometric characteristics in community dwelling Ethiopian adults but some aspects of its validity like dimensionality are still unresolved [7, 10–12]. Therefore, efforts to provide a valid and easy to use questionnaire tool to assess sleep health in Ethiopians students are needed.

The Leeds Sleep Evaluation Questionnaire (LSEQ) is a widely used tool for the diagnosis of sleep disorders including insomnia [13, 14]. The LSEQ was developed to monitor sleep changes during psychopharmacological investigations [14–16]. Available evidence indicates that the LSEQ can be adapted for application in non-pharmacological settings [17]. The psychometric properties of the LSEQ have not been investigated in the African population including Ethiopians. The present study therefore sought to validate the adapted version of the LSEQ (LSEQ-M) in a sample of Ethiopian university students.

## Methods

A sample of 750 students was selected by simple random sampling method across Mizan campus of the Mizan-Tepi University, Mizan-Aman town, Bench Maji Zone, Southwest, Ethiopia. Four hundred and twenty four completed the cross-sectional study i.e. provided filled in answers for LSEQ, Generalized Anxiety Disorder Scale-7 (GAD-7), sub-structured questionnaire for socio-demographics, and participated in a clinical interview. The majority of the participants were males (82.5%), and young adults (age = 21.87 ± 4.13 years, and body mass index = 20.84 ± 3.18 kg/m^2^). Self-reported problems with memory was the exclusion criteria. The purpose and procedures of the study were explained to the volunteers in detail. The university students comprised of many ethnicities, some of them had limited reading proficiency level of the national language i.e. Amharic [7]. Therefore, the modified version of the tool called LSEQ-Mizan (LSEQ-M) (Additional file 1) and the original version of GAD-7 were administered in English by the instructor to the participants [18].

The LSEQ is composed of10 self-reported items each of which is scored on 100 mm visual analogue scale. These items are related to the ease of getting to sleep (GTS), quality of sleep (QOS), ease of awakening from sleep (AFS) and alertness and behavior following wakefulness (BFW) [14]. The items were adapted and modified to make it suitable for screening of sleep problems in university students. This adapted and modified English version of the LSEQ used is referred as LSEQ-Mizan (LSEQ-M). ‘Usual’ was replaced with ‘normal’ from questions related to the GTS and the AFS in the LSEQ-M compared to LSEQ. ‘More difficult than usual’, ‘easier than usual’, ‘slower than usual’, ‘more quickly than usual’, ‘I feel less sleepy than usual’, ‘more sleepy than usual’ were replaced by ‘difficult’, ‘easier’, ‘slower’, ‘more quicker’, ‘less sleepy, and ‘more sleepy’ respectively in the three items of the GTS in the LSEQ-M compared to the LSEQ. The phrase ‘than usual’ was deleted from the two items of the QOS and last item of the BFW in the LSEQ-M compared to the LSEQ. ‘More difficult than usual’, ‘easier than usual’, ‘requires a period of time longer than usual’, ‘shorter than usual’ were replaced by ‘more difficult’, ‘easier’, ‘requires longer period of time’, and ‘requires shorter period of time’ respectively in the two items of the AFS in the LSEQ-M compared to the LSEQ. The reported score for each item was divided by 10 to get an individual item score between 0 and 10. Such scores (between 0 and 10) for each item were added to get LSEQ-M global score with a range of 0-100. EFA in our sample did not support the original 4-factor structure; therefore, we did not adopt the original scoring guideline [16]. In our adapted LSEQ-M, on each item of 100 mm visual analogue, 0 indicated worst sleep condition and 100 suggested normal state. The visual analog scale in the LSEQ-M was marked at intervals of 10 unlike the original LSEQ. Therefore, lower scores of the adapted LSEQ-M indicated poor sleep.

An experienced sleep researcher blinded to the LSEQ-M score clinically interviewed all the participants who completed the study. The presence of insomnia was determined according to the International Classification of Sleep Disorders, revised criteria (ICSD-3) [7, 19]. These criteria included: (i) Insufficient amount of sleep almost every night, (ii) Feeling of restlessness after usual sleep and (iii) At least mild impairment of social or occupational functioning, (iv) Self-reported restlessness, irritability, anxiety, daytime fatigue, and tiredness. The students were classified as insomniacs if they had either of the first two conditions (i.e. i or ii), and at least mild complaints related to both (iii) and (iv) [1, 19]. LSEQ-M has been found to be a valid and reliable measure of insomnia in French and Israeli populations [14].

### Statistical analysis

The statistical analysis was performed using SPSS version 16.0 (SPSS Inc., Chicago, USA) along with AMOS (Analysis of Moment Structures, an add-on module). Internal consistency was assessed by the Cronbach alpha test, while internal homogeneity was tested by Pearson’s correlation analysis between LSEQ-M items and the LSEQ-M global scores. Discriminative validity was assessed by independent t-test for LSEQ-M item as well as the LSEQ-M global score. Diagnostic validity was evaluated by receiver operating curve (ROC) analysis. ICSD-3 based screening for primary insomnia by sleep expert served as the gold standard and the LSEQ-M global score was the test variable [19]. Area under the curve (AUC), cut off score, sensitivity and specificity were assessed.

Multivariate outliers were estimated by calculation of Mahalanobis distance (criterion of a = .001 with 10 df (number of variables), the critical Χ^2^ = 29.59). Twenty two outliers were deleted for factor analysis with Χ^2^ > 29.59 [20]. There was no issue of multicollinearity and singularity; high value of collinearity index of Tolerance and Condition Indexes less than 30 [21]. None of the items were skewed (Skewness z < ±3.29); however, all were platykurtic (Kurtosis z > 3.29). Nevertheless, as the LSEQ-M is an established tool, no deletion or transformations of items was performed [21]. Exploratory factor analysis (EFA) was performed using Principal Axis Factoring extraction and direct oblimin rotation method.

Confirmatory factor analysis (CFA) was performed using maximum-likelihood extraction. The factor loadings (standardized estimates) of the LSEQ-M items on the latent factors were calculated. The CFA was run on six models of the LSEQ-M (Table 7); 1-Factor, 2-Factor correlated, 2-Factor uncorrelated, 4-Factor correlated, second order: 2-Factor, and second order: 4-Factor. Multiple fit indices from different categories; Goodness of fit index (GFI), Adjusted goodness of fit index (AGFI), Comparative Fit Index (CFI), root mean square error of approximation (RMSEA), expected cross-validation index (ECVI) and Chi square statistics were determined. These helped to evaluate the absolute adequate fit, as well as the relatively better fit of the models [20].

## Results

The socio-demographics of the Ethiopian university students participating in the study are given in Table 1. The mean LSEQ-M total score was 58.31 ± 21.49, and the prevalence of primary insomnia was 31.4%. The vast majority of participants reported the habit of tea/coffee consumption (91.7%), beverage intake (59.7%) and class attendance above 90% (77.36%) (Table 1). Table 2 shows the item analysis of the LSEQ-M in the study population. The presence of ceiling or floor effect was scored if more than 15% of respondents reported the highest or lowest score, respectively [7, 22, 23]. Overall, the LSEQ-M total score did not have floor and ceiling effects; 0.9% of Ethiopian university students reported a minimum score of zero, and 7.5% reported a maximum score of 100. Only Item-9 showed floor effect but ceiling effect was observed for all the ten items [7, 22, 23]. The internal consistency test of the LSEQ-M showed a Cronbach’s alpha of 0.84, a value indicating good consistency. The internal homogeneity as shown by Pearson’s correlation coefficient (r) between item scores and the LSEQ-M total score was 0.60-0.69. All the correlation coefficients were significant (*p* < 0.01) (Table 3).

**Table 1:** Socio-demographics of Ethiopian university students

Characteristics	Mean ± SD/ Frequency
Age (yr)	21.87 ± 4.13
BMI (Kg/m^2^)	20.84 ± 3.18
Gender	
Male	350(82.5%)
Female	74(17.5%)
Ethnicity	
Bench	33(7.8%)
Kaffa	15(3.5%)
Oromo	129(30.4%)
Amhara	139(32.8%)
Tigre	3(0.7%)
Wolaita	8(1.9%)
Others	97(22.9%)
Religion	
Orthodox Christian	208(49.1%)
Protestants Christian	136(32.1%)
Catholic	1(0.2%)
Islam	70(16.5%)
Others	9(2.1%)
Years of university education	
1 yr	152(35.8%)
2 yr	153(36.1%)
3 yr	48(11.3%)
4 yr	38(9.0%)
5 yr	33(7.8%)
Attendance	
Upto 80%	54(12.74%)
80-90%	42(9.91%)
90-100%	328(77.36%)
Monthly Family Income (In Birr)	
Very Low (less than 445)	44(10.4%)
Low (446-1200)	78(18.4%)
Average (1201-2500)	55(13.0%)
Above average (2501-3500)	30(7.1%)
High (greater than 3500)	80(18.9%)
Unknown	137(32.3%)
Parents	
Single	180(42.5%)
Married	236(55.7%)
Divorced	8 (1.9%)
GAD-7	7.24 ± 4.47
Sleep	
LSEQ score	58.31 ± 21.49
ICSD-R Classification	
Primary insomnia/normal	133 (31.4%)/ 291 (68.6%)
Substance use/ Habits	
Chat user/non-user	18(4.2%)/406(95.8%)
Alcohol user/alcohol non-user	23(5.4%)/401(94.6%)
Smoker/non-smoker	2(0.5%)/422(99.5%)
Tea/Coffee consumer/ non-consumer	389 (91.7%)/35(8.3%)
Beverage consumer/beverage non-consumer	253(59.7%)/171(40.3%)

*BMI* Body mass index; LSEQ, *ICSD-R* International Classification of Sleep Disorders, revised criteria, *GAD-7* Generalized Anxiety Disorder-7

**Table 2:** Descriptive statistics of the Leeds Sleep Evaluation Questionnaire (LSEQ) in Ethiopian university students

Leeds Sleep Evaluation Questionnaire (LSEQ) items	Mean ± SD	Skewness		Kurtosis	
		Skewness±SE	z	Kurtosis±SE	z
Getting to sleep item 1	6.10 ± 3.21	−0.38 ± 0.12	−3.08	−0.93 ± 0.24	−3.82
Getting to sleep item 2	5.75 ± 3.21	−0.28 ± 0.12	−2.30	−0.91 ± 0.24	−3.76
Getting to sleep item 3	5.86 ± 3.31	−0.28 ± 0.12	−2.25	−1.07 ± 0.24	−4.40
Quality of sleep item 1	5.88 ± 3.18	−0.17 ± 0.12	−1.35	−1.07 ± 0.24	−4.39
Quality of sleep item 2	5.89 ± 3.30	−0.31 ± 0.12	−2.51	−1.01 ± 0.24	− 4.17
Awake following sleep item 1	5.78 ± 3.21	−0.24 ± 0.12	−1.96	−0.99 ± 0.24	−4.07
Awake following sleep item 2	5.76 ± 3.16	−0.25 ± 0.12	−2.07	−0.95 ± 0.24	−3.91
Behaviour following wakening item 1	5.70 ± 3.35	−0.18 ± 0.12	−1.49	− 1.14 ± 0.24	−4.70
Behaviour following wakening item 2	5.86 ± 3.43	−0.32 ± 0.12	−2.61	−1.14 ± 0.24	−4.67
Behaviour following wakening item 3	6.12 ± 3.32	−0.35 ± 0.12	−2.86	−1.02 ± 0.24	−4.18

*SD* Standard deviation, *SE* Standard Error

**Table 3:** Internal consistency and homogeneity of the Leeds Sleep Evaluation Questionnaire (LSEQ) scores in Ethiopian university students

Items of the LSEQ	Item-to-global LSEQ score correlations	Cronbach’s Alpha if Item Deleted
Getting to sleep item 1	.69^**^	.81
Getting to sleep item 2	.62^**^	.82
Getting to sleep item 3	.62^**^	.82
Quality of sleep item 1	.65^**^	.82
Quality of sleep item 2	.60^**^	.82
Awake following sleep item 1	.67^**^	.82
Awake following sleep item 2	.62^**^	.82
Behaviour following wakening item 1	.63^**^	.82
Behaviour following wakening item 2	.64^**^	.82
Behaviour following wakening item 3	.62^**^	.82

**p < 0.01

**Fig. 1:**
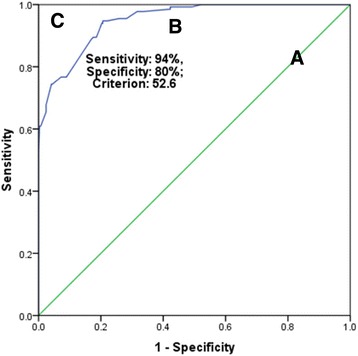
Receiver operator curves (A) No discrimination (AUC = 0.5) (B) Experimental test (0.95 (*p* < 0.001)) and (C) Perfect test (AUC = 1.0) in Ethiopian university students

The groups of Ethiopian university students identified as normal and with moderate anxiety levels based on GAD-7 evaluation differed across the LSEQ-M total score, as well as scores of all the items score except the item-9 (Table 4). The diagnostic validity was assessed by the ROC curve (Fig. 1). The sensitivity and specificity of the LSEQ-M at the cut-off score of 52.6 were 94% and 80%, respectively.

**Table 4:** Discriminative validity: Comparison of the Leeds Sleep Evaluation Questionnaire (LSEQ) scores between Ethiopian university students with normal and moderate anxiety levels

Items of the LSEQ	Mean (SD) score		*t*	*df*	*p*-value
	Normal (*n* = 125)	Moderate anxiety (*n* = 97)			
Getting to sleep item 1	7.13 (3.07)	5.82 (3.07)	3.14	220	.002
Getting to sleep item 2	6.68 (3.32)	4.96 (3.02)	4.01	212.50	.006
Getting to sleep item 3	6.54 (3.46)	5.29 (3.23)	2.76	220	< 0.001
Quality of sleep item 1	7.16 (2.94)	5.42 (3.16)	4.22	220	< 0.001
Quality of sleep item 2	6.56 (3.46)	5.49 (3.42)	2.30	220	.022
Awake following sleep item 1	6.59 (3.34)	4.87 (3.07)	3.94	220	< 0.001
Awake following sleep item 2	6.49 (3.35)	5.15 (3.09)	3.09	213.42	.002
Behaviour following wakening item 1	6.62 (3.42)	4.66 (3.26)	4.31	220	< 0.001
Behaviour following wakening item 2	6.29 (3.63)	5.42 (3.47)	1.80	220	.073
Behaviour following wakening item 3	6.85 (3.31)	5.55 (3.31)	2.89	220	.004
LSEQ total score	66.92 (21.92)	52.65 (19.96)	5.00	220	< 0.001

*Mean ± SD

The sample satisfied the conditions for factor analysis as indicated by the results of Kaiser-Meyer-Olkin test of sampling adequacy, Bartlett’s test of sphericity, anti-image matrix (Table 5) and communality retention criteria (0.37–0.57) (Table 6) [11, 24]. The three tests employed to estimate the number of factors in EFA i.e. Kaiser’s criteria (Eigenvalue > 1), Scree plot and cumulative variance rule (> 40%) found different number of factors. Kaiser’s criteria (Eigenvalue > 1) and Scree plot identified 2-factor model, while cumulative variance rule (> 40%) found 1-factormodel for the LSEQ-M (Table 5). The loadings of the LSEQ-M items in EFA retained for performing CFA ranged from 0.34 to − 0.90 (Table 6).

**Table 5:** Summary of the sample size adequacy measures and exploratory factor analysis of the Leeds Sleep Evaluation Questionnaire (LSEQ) in Ethiopian university students

Measures	LSEQ (10-item scale)
Kaiser-Meyer-Olkin Test of SamplingAdequacy	0.85
Bartlett’s test of Sphericity	< 0.001
Anti-image matrix	0.79-0.91
Determinant	0.02
Number of factors	
Kaiser’s criteria (Eigenvalue> 1)	2
Cumulative variance rule (> 40%)	1
Scree plot	2

*LSEQ* Leeds Sleep Evaluation Questionnaire

**Table 6:** Factor matrix of the 2-Factor model of the Leeds Sleep Evaluation Questionnaire (LSEQ) in Ethiopian university students

Leeds Sleep Evaluation Questionnaire (LSEQ) items	Factor-1^a^	Factor-2^a^	Communality (h2)
Getting to sleep item 1	.57	−.20	.46
Getting to sleep item 2	.72	.04	.45
Getting to sleep item 3	.76	.08	.45
Quality of sleep item 1	.56	−.19	.46
Quality of sleep item 2	.68	.04	.37
Awake following sleep item 1	.02	−.77	.56
Awake following sleep item 2	−.12	−.90	.57
Behaviour following wakening item 1	.08	−.61	.42
Behaviour following wakening item 2	.29	−.38	.42
Behaviour following wakening item 3	.34	−.32	.38

Exploratory Factor analysis (EFA) with Principal Axis Factoring extraction and direct oblimin rotation method was performed

^a^Latent factors derived from EFA

As indicated by significant χ2 *p*-value; none of the models had absolute fit to the data (Table 7). The results of the CFA did not validate the models indicated by EFA. The original 4-Factor correlated model performed best with lowest values for RMSEA, χ2/df, χ2, and ECVI, while it showed highest values for GFI, AGFI and CFI (Table 7).

**Table 7:** Fit statistics of the Leeds Sleep Evaluation Questionnaire (LSEQ) in Ethiopian university students

Models	GFI	AGFI	CFI	RMSEA	χ^2^	df	p	χ^2^/df	ECVI
1-Factor	.88	.81	.81	.12 (.11-.14)	267.17	35	<.001	7.63	.73
2-Factor correlated	.92	.87	.87	.10 (.09-.12)	187.39	34	<.001	5.51	.54
2-Factor uncorrelated	.87	.81	.75	.14 (.13-.16)	342.68	35	<.001	9.79	.91
4-Factor correlated	.94	.89	.92	.09 (.07-.10)	121.50	29	<.001	4.19	.41
Second order: 2-Factor	.92	.87	.87	.10 (.08-.12)	187.39	34	<.001	5.51	.54
Second order: 4-Factor	.93	.88	.91	.09 (.08-.11)	145.16	31	<.001	4.68	.46

*GFI* Goodness of fit index, *AGFI* Adjusted goodness of fit index, *CFI* Comparative Fit Index, *RMSEA* root mean square error of approximation, *ECVI* ECVI expected cross-validation index

## Discussion

This is the first study to examine the psychometric and diagnostic validity of the modified English version of the LSEQ in a non-pharmacological setting. In this study, the LSEQ-M was validated in Ethiopian university students using ICSD-R criteria for screening of insomnia. The individual items of the LSEQ-M had ceiling and floor effects but the LSEQ-M global score did not have either of these effects (Table 2). Thus, item analysis does support validity of the overall score of the scale [22]. Our findings did not show either ceiling or floor effects for the LSEQ-M. Similarly, neither ceiling nor floor effects were observed in the Korean version of the LSEQ global score [17].

The Cronbach’s alpha test showed that the scale had good internal consistency in this population of Ethiopian university students. It is comparable to values reported in the French and Israeli insomniacs [14]. Tarrasch et al. [14] reported Cronbach’s alpha values (0.78-0.92) for factors of the original 4-Factor model of the LSEQ. However, Kim et al. reported excellent value of Cronbach alpha (.95) in Korean older adults [17]. There were little changes in Cronbach’s alpha test if items were deleted suggesting almost similar relevance of items in the LSEQ-M construct (Table 3). The item-LSEQ-M global score had strong correlations (Table 3). Moreover, the close range of correlations suggests that all the 10 items are almost equally relevant for construct of the scale. This is unlike the case with PSQI, in which some items are less sensitive in particular populations [4, 7, 25]. Therefore, internal consistencies as well as internal homogeneity favor validity of the LSEQ-M over the PSQI in Ethiopians [7].

The significantly lower values of the LSEQ-M global as well as all the items (item-9) among those with moderate level of anxiety as measured by the GAD establish the diagnostic known-group or discriminative validity of the tool in this population of Ethiopian university students (Table 4). Insomnia has been shown to be associated with anxiety disorder [26]. Notably, with regard to discriminative validity as well, the LSEQ-M has favorable validation than the PSQI in Afro-Asian populations [1, 4, 7].

The diagnostic validity of the scale against ICSD-3 criteria for insomnia in this sample of Ethiopian university students was in an excellent range [27]. Few studies have investigated the AUC of the LSEQ. The AUC of 0.95 (CI: 0.93-0.97) (Fig. 1) found in our study was higher than that reported in Korean older adults i.e. 0.86 (95% CI: 0.83-0.90) [17]. Unlike our use of ICSD-3, Kim et al... had employed Insomnia Severity Index as the concurrent measure [17]. In Kim et al study the cut-off score was 66.5. However, as we had adopted a reverse scoring, the effective value of cut-off score from their study based on the reverse scoring will be 33.5 (100-66.5). Therefore, a cut-off score (52.6) (Fig. 1) for screening insomnia in our study sample of Ethiopian university students was higher than that reported in Korean older adults [17]. The accuracy (89%) of the LSEQ-M at the cut-off score was higher than that reported in the Korean study [17]. This suggests that the LSEQ-M that was used in our study on Ethiopian university students had favorable diagnostic validity than the modified LSEQ used in Korean older adults [17]. The accuracy of the LSEQ-M in this study sample is also higher than the accuracy reported for the PSQI in Ethiopians [7]. Therefore, the PSQI is probably only another sleep tool to be validated in Ethiopians for screening of insomnia as per ICSD-R criteria [7].

The results of the EFA were inconclusive, but the outcome of CFA favored the original 4-Factor model of the LSEQ-Min the Ethiopian university students (Tables 5, 6 and 7). The three factors in EFA suggested heterogeneity of the LSEQ factor structure (Tables 5 and 6). However, the original 4-Factor model of the LSEQ-M showed highest values for GFI, AGFI, CFI, and least values for χ2, χ2/df, RMSEA and ECVI (Table 7). This favored the validity of the original 4-Factor model over all other models tested [11, 16, 24].

Biased gender ratio in the study sample and non-application of objective measurement of sleep i.e. polysomnography, actigraphy are important limitations. The gender ratio in the study sample was 0.22, although it is 0.55 in the university students. Female students were less likely to complete the clinical interview, which might have resulted in gender bias. The test re-test reliability and inter/intra-rater reliability were not assessed. Future studies should look into this aspect. The merits include validation of a tool in a population, which has high prevalence of sleep problems but does not have access to advanced sleep medicine professionals and/or facilities.

## Conclusion

The study findings suggest that LSEQ has favorable psychometric validity than the PSQI in Ethiopians. The LSEQ-M was found to be a valid tool for screening for insomnia in this sample of Ethiopian university students.

## Additional file

Additional file 1: (SAV 30 kb)
